# Personalized Ultra-Fractionated Stereotactic Adaptive Radiotherapy for Non-Small Cell Lung Cancer Using Varian Ethos Therapy System

**DOI:** 10.3390/curroncol31120562

**Published:** 2024-12-01

**Authors:** Vanda Leipold, Blanka Jakšić, Asmir Avdičević, Domagoj Kosmina, Hrvoje Kaučić, Ivana Alerić, Karla Schwarz, Mihaela Mlinarić, Giovanni Ursi, Adlan Čehobašić, Dragan Schwarz

**Affiliations:** 1 Specialty Hospital Radiochirurgia Zagreb, 10431 Sveta Nedelja, Croatiadomagoj.kosmina@radiochirurgia.hr (D.K.);; 2 Faculty of Medicine, Josip Juraj Strossmayer University of Osijek, 31000 Osijek, Croatia; 3 School of Medicine, University of Zagreb, 10000 Zagreb, Croatia; 4 Faculty of Medicine, Juraj Dobrila University of Pula, 52100 Pula, Croatia

**Keywords:** NSCLC, personalized ultra-fractionated stereotactic adaptive radiotherapy, adaptive radiotherapy, Varian Ethos, HyperSight

## Abstract

We present a patient treated with personalized ultra-fractionated stereotactic adaptive radiotherapy (PULSAR) for non-small cell lung cancer (NSCLC) using the adaptive Varian Ethos™ system equipped with the novel HyperSight imaging platform. Three pulses of 12 Gy were separated by a pause of four weeks during which the tumor was given enough time to respond to treatment. Only initial planning computed tomography (CT) was acquired on a CT simulator (Siemens Somatom Definition Edge), whereas other pulses were adapted using online cone beam computed tomography (CBCT) images (iCBCT Acuros reconstruction) acquired while the patient was lying on the treatment couch and delivered immediately. Significant tumor reduction was achieved between pulses, resulting in improved organs-at-risk sparing. In addition, the on-couch plan optimization based on CBCT greatly reduced the patient’s stay at the clinic and the duration of treatment preparation.

## 1. Introduction

Conventionally fractionated adaptive radiotherapy has been used to manage anatomical changes for patients with non-small cell lung cancer (NSCLC), such as tumor shrinkage or atelectasis [1,2,3]. This approach allows for better organs-at-risk (OARs) sparing and dose escalation to the tumor, thus improving therapeutic outcomes for patients with locally advanced NSCLC [4].

Personalized ultra-fractionated stereotactic adaptive radiotherapy (PULSAR) is a novel approach to stereotactic body radiotherapy, where instead of following standard fractionation schemes, patients are treated in pulses. Every pulse (usually 8 Gy or more) is followed by a pause (several weeks to several months), during which the tumor is given time to respond to treatment; due to the high pulse dose, the tumor is not likely to progress during the pause [5]. After the pause, a new planning CT image is acquired, and a new plan, adapted to the changes in tumor volume, is generated. Functional imaging or biopsy can also be performed to acquire additional information on tumor response. The patient is then treated using the new plan followed by another pause, after which the process of tumor response information acquisition, imaging, and adaptive plan generation is repeated. As the tumor regresses during the pauses, overtreatment can be avoided and treatment outcomes improved by sparing healthy tissue, potentially leaving room for future treatment or dose escalation [6,7,8,9,10].

Historically, adaptation required a new simulation CT and the generation of a new plan to account for anatomical changes such as a reduction in tumor size. However, recent advances in imaging technology and artificial intelligence [11,12,13,14] have allowed for daily adaptive treatments based on on-couch imaging. Immediately after the new CBCT images are acquired (while the patient is still lying on the couch), a new adapted plan is generated, evaluated, and delivered. This approach greatly reduces the treatment preparation time as well as the number and duration of the patient’s stays at the hospital.

The image quality of the HyperSight CBCT system, specifically iCBCT Acuros reconstruction, has been well documented in the literature [15,16,17]. CT calibration curves for CT number and relative electron density based on electron density phantom measurements for both the full CT scan and iCBCT Acuros show a closely matching CT number calibration error for iCBCT Acuros (1.06%) compared to CT scanner (0.72%) [17]. This is in agreement with our investigations. The analysis of DVH parameters for OAR and PTV in the case of the prostate shows agreement within 2% between fan-beam CT and CBCT [16], and dosimetric comparison using gamma analysis of treatment plans recalculated on CBCT has a gamma passing rate of more than 93% when compared to plans on CT, using a 20% dose threshold and 2%/1 mm gamma criteria [15]. The CBCT data set for planning in the Ethos environment is combined with a synthetic CT to account for the lack of scatter data in the longitudinal direction due to the field of view limitations of the CBCT acquisition.

## 2. Patient and Methods

A 63-year-old Caucasian female patient was admitted for a diagnostic work-up due to an accidental finding of a right lung lesion on a chest X-ray. Although the patient did not experience any pulmonary symptoms (e.g., dyspnea, cough, or chest pain), she was a heavy smoker with a medical history of chronic obstructive pulmonary disease. A fibrobronchoscopy was performed, which revealed a large tumor infiltration of the bronchial mucosa in the bronchus of the right upper lung segment with complete obstruction of the right secondary bronchus. During this procedure, transbronchial needle aspiration and a biopsy were performed. A cytology specimen showed non-small malignant cells, possibly adenocarcinoma, and a histology specimen showed carcinoma of planocellular differentiation. The specimens were EGFR negative and PD-L1 positive (>90%). CT scans showed a lobulated lesion in the right upper lung segment measuring 39 × 29 × 37 mm, infiltrating some segments of pulmonary artery branches with a few satellite nodular lesions. There was no enlargement of the mediastinal lymph nodes, nor signs of disease dissemination. According to current guidelines, pathologic evaluation of mediastinal lymph nodes must be carried out—including mediastinoscopy, mediastinotomy, endobronchial ultrasound (EBUS), endoscopic ultrasound, and CT-guided biopsy—in order to exclude mediastinal involvement. Therefore, EBUS with transbronchial node biopsy was performed. The histology specimens were negative for malignancy. Fluorodeoxyglucose positron emission CT imaging revealed no signs of lymph node involvement or distant metastasis. The disease was staged as IIB (cT3N0), and the patient was presented to thoracic surgeons, according to whom the patient was medically inoperable due to poor pulmonary status. The multidisciplinary team decided to treat the patient using stereotactic ablative radiotherapy. Neither concurrent chemoradiotherapy nor immunotherapy was considered due to the poor pulmonary function. CT scans performed for simulation purposes showed enlargement of the intrapulmonary lesion, which measured 71 × 49 mm at that time. Our multidisciplinary team indicated PULSAR to treat the NSCLC using the adaptive Varian Ethos™ system.

To generate adapted plans using the Ethos system, an initial simulation and plan had to be made. This initial plan (also called a reference plan) was used as an optimization and contouring template for subsequent automatically generated online adapted plans.

Unlike online adapted plans, the generation of a reference plan normally takes significantly longer (several hours in this case) and thus could not be conducted online while the patient was waiting on the couch. Since the patient was treated in free breathing, a 4DCT was necessary to obtain information on the tumor respiratory movement needed for ITV volume definition. Initial planning CT images (free breathing, average, and 4DCT) were acquired using a Siemens Somatom Definition EDGE scanner.

Between all 10 phases of breathing, maximum lesion excursions did not exceed 3 mm, 5 mm, and 1 mm in the anterior-posterior, craniocaudal, and lateral directions, respectively.

Since a 4DCT could not be acquired during online adaptation, adapted planning was performed using a free-breathing CBCT, and a clinical target volume (CTV) structure was generated. Since HyperSight CBCT acquisition typically only lasts 6 s, it underestimates lesion respiratory movement and usually corresponds to an exhale breathing phase. Therefore, CTV to internal target volume (ITV) margins corresponding to 4DCT-detected maximal lesion excursions from the exhale phase (3 mm, 5 mm, and 1 mm in the anterior, caudal, and right direction, respectively) were added.

Finally, the ITV was expanded with an additional isotropic margin of 5 mm to generate a PTV.

The principal OARs were the lung, heart, tracheobronchial tree, and the first, second, and third right ribs. An adapted volumetric modulated arc therapy plan with three arcs and a total peripheral dose to the tumor of 36 Gy (D 50% = 45 Gy) in three fractions was generated using the Ethos treatment planning system (TPS) version 02.01.00 (Varian Medical Systems) and exported to Varian Eclipse TPS for the purposes of treatment comparison, as well as to the Mobius second-check dosimetry system for plan evaluation. Patient intrafraction motion was monitored using the AlignRT (VisionRT, London, UK) patient surface monitoring system. To optimize and calculate adapted plans, a HyperSight^TM^-acquired CBCT with iCBCT Acuros reconstruction was used. Each pulse was followed by a pause of four weeks, allowing for tumor response and reduction in volume.

After the pause, a new adapted plan was generated in the same manner, using CBCT images acquired while the patient was lying on the treatment couch waiting for the treatment.

## 3. Results

**Figure 1:**
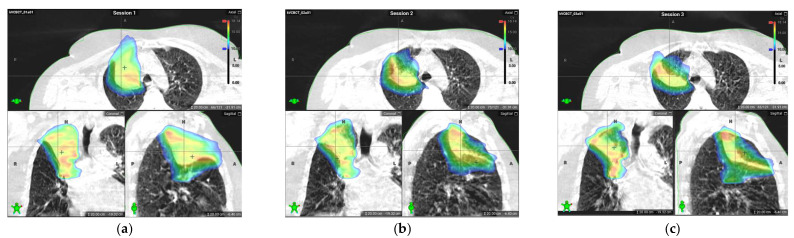
Comparison of dose distributions delivered during the first, second, and third pulses: (**a**) dose distribution delivered based on cone beam computed tomography (CBCT) images acquired during the first pulse; (**b**) dose distribution delivered based on the CBCT image acquired during the second pulse, four weeks after first pulse; (**c**) dose distribution delivered based on the CBCT image acquired during the third pulse, eight weeks after the first pulse. A substantial reduction in lesion size and dose to the surrounding tissue can be seen.

A substantial reduction in tumor volume resulted in a smaller ITV and a reduced dose to the surrounding organs, as seen in Figure 1.

In-house three-fraction volume dose constraints to the ribs (volume receiving 28.8 Gy should not exceed 1 cm^3^), tracheobronchial tree (volume receiving 15 Gy should not exceed 4 cm^3^), and lung (volume receiving 20 Gy should not exceed 10% and must be less than 20 of the lung) were used in plan optimization and evaluation. Since the total dose was delivered in three pulses, volumes of OARs receiving one-third of the constraint dose are reported and compared between pulses, as shown in Table 1.

Maximum doses (listed as doses delivered to 0.1% of the volume of the OAR) are also compared between pulses for the tracheobronchial tree, aorta, and pulmonary artery, as shown in Table 2.

The reduction in target volume after the pulses (followed by pauses) resulted in a significant decrease in the dose to healthy tissue for all tissues except for the lungs. Due to tumor shrinkage, more lung tissue in the apical segment was exposed to a high dose when comparing the second and third pulse with the first one.

Reference plan generation alone (not including initial simulation and contouring) took several hours for this patient. The time required to generate online adaptive plans is significantly shorter, as shown in Table 3. In fact, the whole treatment simulation, plan generation, and delivery of an adapted plan lasted less time than the generation of the reference plan.

In addition, the patient had to travel to the hospital only for the initial CT simulation, since the others were carried out as a part of the online adaptation. This further accelerated and simplified the entire procedure.

## 4. Discussion

In our case, the initial and second pulses resulted in a significant ITV reduction of 18% and 28%, leading to better overall OAR sparing during the second pulse, as observed by Moore et al. [6], Sezen et al. [7], and Rahimian et al. [9].

These findings are in accordance with previous PULSAR treatments delivered in our institution using the Varian Edge system. The change from Edge to Ethos adaptive systems reduced both the number and duration of patient stays at the hospital, as only the initial pulse requires a separate simulation CT and offline optimization process. Thus, the delay between planning CT acquisition and treatment delivery was reduced to a minimum. The whole duration of a pulse treatment, from the patient’s arrival to iCBCT acquisition, online plan adaptation, and treatment delivery, lasted no more than 65 min (47, 65, and 63 min for the first, second, and third pulse, respectively).

## 5. Conclusions

This report reinforces the previously stated concern that the treatment of large NSCLC in consecutive fractions without pauses and adaptations could result in overtreatment and increased toxicity compared with PULSAR. In addition, it demonstrates that an intelligent adaptive Ethos platform can reduce the number and duration of PULSAR patient stays at the hospital, as well as the time spent by staff (i.e., no need for separate additional CT simulations, as each new pulse is adapted based on an iCBCT acquired on the treatment couch). The Ethos intelligent adaptive system appears to be a promising platform for personalized treatments such as PULSAR.

## Figures and Tables

**Table 1:** Target structure volumes and organ at risk (OAR) volumes receiving one-third of the constraint doses (since the treatment was delivered in three pulses) compared between the pulses.

Target Structures, OAR Structures (Third of the Constraint Dose)	First Adapted Pulse (cm^3^)	Second Adapted Pulse (cm^3^)	Third Adapted Pulse (cm^3^)
ITV	207.5	170.0 (−18%)	149.6 (−28%)
PTV	323.3	287.6 (−11%)	275.7 (−15%)
Rib 1 (9.6 Gy)	7.5	3.2 (−57%)	4.2 (−44%)
Rib 2 (9.6 Gy)	6.7	4.2 (−37%)	4.6 (−31%)
Rib 3 (9.6 Gy)	0.9	1.6 (+78%)	2.3 (+156%)
Tracheobronchial tree (5.5 Gy)	1.8	1.3 (−28%)	1.2 (−33%)
Lungs (6.7 Gy)	381.0	400.3 (+5%)	425.9 (+12%)

**Table 2:** Maximum doses to organs at risk (OARs) (doses delivered to 0.1% volume of the organ) compared between three pulses.

Target Structures, OAR Structures (Third of the Constraint Dose)	First Adapted Pulse (Gy)	Second Adapted Pulse (Gy)	Third Adapted Pulse (Gy)
Aorta	12.2	10.1 (−17%)	10.7 (−12%)
Pulmonary artery	14.2	13.1 (−8%)	12.4 (−13%)
Tracheobronchial tree	9.8	8.6 (−12%)	8.5 (−9%)

**Table 3:** Time spent at each step of the treatment, from patient arrival to planning CBCT acquisition, through contour and plan generation, to plan delivery.

	Procedure Duration		
Time Intervals	First Adapted Pulse (min)	Second Adapted Pulse (min)	Third Adapted Pulse (min)
From patient arrival to planning CBCT acquisition	6	16	6
From contour and plan generation to plan acceptance	20	31	42
First control CBCT and partial treatment delivery	11	7	7
Second control CBCT and the remaining treatment delivery	10	11	8
Total treatment time	47	65	63

## Data Availability

The data generated for this study are available in this article or from the corresponding author upon request.
